# Causal associations among gut microbiota, 1400 plasma metabolites, and asthma: a two-sample Mendelian randomization study

**DOI:** 10.3389/fmolb.2024.1370919

**Published:** 2024-07-22

**Authors:** Lizhu Wang, Zhe Lv

**Affiliations:** ^1^ Guizhou University of Traditional Chinese Medicine, Guiyang, China; ^2^ Air Force Medical University Tangdu Hospital, Xi’an, China

**Keywords:** Mendelian randomization, intestinal flora, asthma, plasma metabolites, causality

## Abstract

**Background:**

Emerging evidence indicates a correlation between imbalances in intestinal microbiota and changes in plasma metabolites in the progression of asthma. However, the causal link between these factors remains unclear.

**Methods:**

A two-sample Mendelian randomization (MR) study was employed to evaluate the potential causal connection between gut microbiota, plasma metabolites, and asthma susceptibility. Gut microbiota data from expansive genome-wide genotype studies and 16S fecal microbiome datasets were examined by the MiBioGen Alliance. Asthma data were procured from the FinnGen biobank analysis, while comprehensive Genome-Wide Association Studies (GWAS) summary statistics for plasma metabolites were derived from the NHGRI-EBI GWAS Catalog. Fluctuations in intestinal flora and plasma metabolites in asthma patients were evaluated using the weighted mode method. Additionally, pleiotropic and heterogeneity analyses were performed to ascertain the reliability of the findings.

**Results:**

Upon examining the gut microbiota through MR with the IVW method, alongside tests for heterogeneity and pleiotropy, findings reveal a negative association between the abundance of the Christensenellaceae R.7 group and asthma risk. In contrast, the Bifidobacterium and Prevotella 7 genera exhibit a positive association with asthma risk, indicating they may be potential risk factors (*p* < 0.05). Furthermore, MR analysis of 1,400 metabolites employing Weighted median, IVW, and Weighted mode methods resulted in *p*-values below 0.05. Subsequent tests for pleiotropy and heterogeneity showed that the levels of 3,5-dichloro-2,6-dihydroxybenzoic acid have a negative correlation with asthma, whereas the phenylalanine to phosphate ratio has a positive correlation, suggesting their potential as risk factors for asthma (*p* < 0.05).

**Conclusion:**

The current Mendelian randomization study provides evidence supporting a potential causal link between specific gut microbiota taxa, plasma metabolites, and asthma. These findings offer novel perspectives for future research and the development of treatment and prevention strategies for asthma.

## 1 Introduction

Asthma, also known as bronchial asthma, is a chronic inflammatory condition that impacts the airways. It is marked by intermittent airflow blockage, severe symptoms, increased bronchial sensitivity, and inflammation (John, 2015; Gisell Mosnaim Asthma in Adults, 2023). Studies have revealed that over 300 million individuals worldwide have asthma, with prevalence increasing rapidly in recent years attributed to air pollution and climate change (Wang et al., 2023). As a major global health issue, asthma imposes significant economic costs on individuals and societies.

Host-microorganism interactions locally modulate cellular functions, affect immune responses, and significantly impact disease progression. Multiple studies have demonstrated that the host-microorganism interactions impact local and peripheral tissues (Marsland et al., 2015). In 1980, McDermott and Bienenstock introduced the concept of a “common mucosal immune system”, suggesting an interconnected network across the respiratory, gastrointestinal, oral, and urogenital tracts. This system, composed of tissues, cells, and effector molecules, aims to defend against pathogens. Further, they proposed that the immune cells within this network facilitate cross-communication between different mucosal tissues (McDermott et al., 1980). Growing evidence supports that stimulating the common mucosal immune system in one area can significantly affect another (Tulic and Piche, 2016). Additionally, research indicated that changes in the intestinal microenvironment, including shifts in microbiota composition, are associated with the progression of various lung diseases, suggesting substantial interaction between the intestinal and respiratory mucosal areas (Bruzzese et al., 2014).

Over the last decade, human microbiome studies have shown that our intestinal symbiote plays a crucial role in maintaining bodily balance by modulating immune responses in the gastrointestinal system and beyond (ckhed et al., 2005). Imbalances in intestinal flora can influence lung diseases by altering immune, hormonal, and metabolite balance (Budden et al., 2016). Numerous studies have established a close relationship between plasma metabolites and asthma (Lumia et al., 2011; Chiu et al., 2021; Ekstr ö m et al., 2022). However, the existence of an interactive axis involving gut microbiota, plasma metabolites, and asthma still requires further investigation.

Mendelian Randomization (MR) is a statistical method that combines data from Genome-Wide Association Studies (GWAS) and uses Single Nucleotide Polymorphisms (SNPs) as instrumental variables to draw causal inferences without confounders and biases (Greenland, 2018). Two-sample MR analysis synthesizes SNP exposure and outcome data from distinct GWAS to produce a comprehensive causal estimate (Liu et al., 2023). In this investigation, two-stage MR analysis was employed to explore the relationship between plasma metabolites in GM and 1400 and asthma. This method enhances our understanding of the relationship, providing a theoretical foundation for asthma research and identifying potential innovative treatment strategies.

## 2 Materials and methods

### 2.1 Study design

**FIGURE 1:**
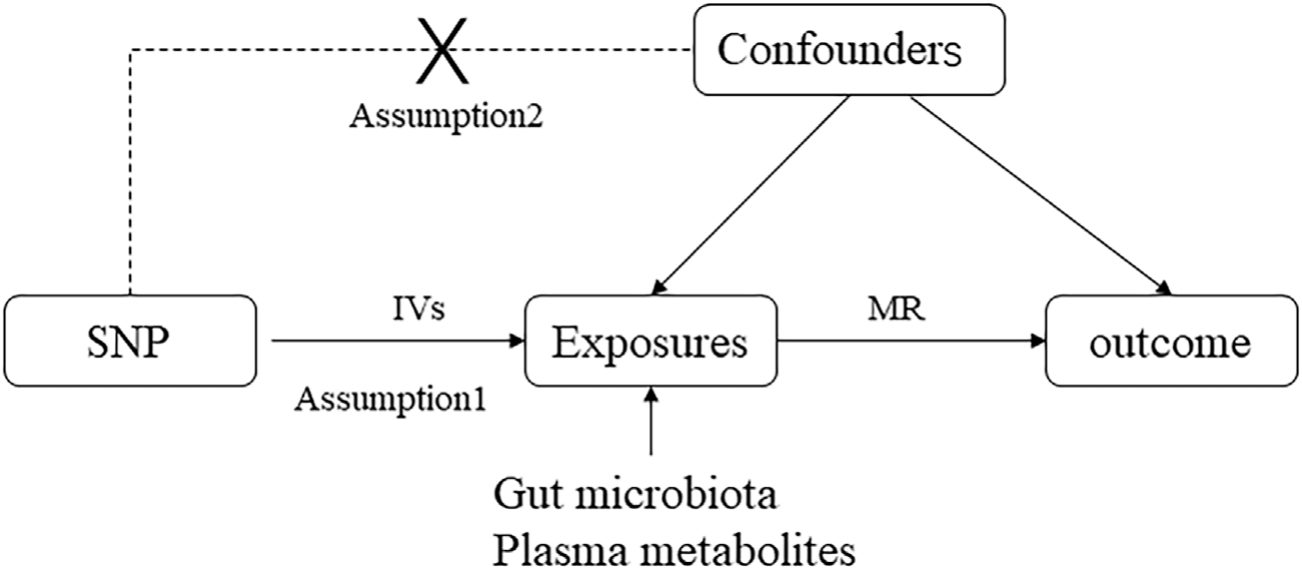
Directed acyclic graphs are employed in classical Mendelian randomization designs. Arrows represent causal relationships between variables, pointing from the cause to the effect. Placing “X” on the arrowed line blocks the causal pathway. MR denotes Mendelian randomization.

This study employed a two-way MR analysis to discern causal relationships among gut microbiota, plasma metabolites, and asthma, as depicted in Figure 1. This analysis treated gut microbiota and plasma metabolites as exposures, while asthma was considered the outcome. GWAS data for gut microbiota were obtained from the Mibiogen consortium, and plasma metabolite data were derived from the comprehensive metabolite statistics in the NHGRI-EBI GWAS Catalog (Chen et al., 2023). Data on asthma were acquired from the FinnGen research project. Genetic variants from these datasets were utilized as instrumental variables (IVs) in two-sample MR analyses conducted using the “TwoSampleMR” package in R software. The study adhered to three fundamental assumptions for MR analyses: (1) a significant correlation between IVs and exposure factors, (2) no correlation between IVs and any confounding factors associated with both exposure and outcome and (3) the exclusive influence of IVs on outcomes via exposures (Skrivankova et al., 2021). All datasets utilized in this study are publicly available, and ethical approval was obtained from the corresponding institutions for each GWAS involved.

### 2.2 Data sources

The study examined 16S ribosomal RNA gene sequencing and genotyping data across 24 populations from nations including the United States, Canada, Israel, Korea, Germany, Denmark, the Netherlands, Belgium, Sweden, Finland, and the United Kingdom. Our team compiled data on 211 bacterial traits across 9 species, 131 genera, 16 classes, 20 orders, and 35 families, excluding undefined taxa (Kurilshikov et al., 2021). The resulting GWAS summary statistics for all 1,091 blood and 309 plasma metabolites are available for download at the NHGRI-EBI GWAS Catalog (https://www.ebi.ac.uk/gwas/). Additionally, GWAS summary data, including statistics for asthma, were obtained from the FinnGen study involving 53,598 asthma cases and 409,335 control subjects of European ancestry, identified in Dataset: ukb-b-18113.

### 2.3 Instrumental variables

This study analyzed the association between asthma as the outcome and gut microbiota and plasma metabolites as exposure variables. IVs were selected following specific guidelines. Initially, SNPs linked to the exposure taxa had to reach a genome-wide significance level (p < 1e-05). Subsequently, a linkage disequilibrium (LD) analysis (R2< 0.001, clustering distance = 10,000 kb), based on the European 1,000 Genome Project, was conducted. SNPs that did not adhere to these criteria were excluded. A statistical value F > 10 was the threshold for identifying an IV as strong. IVs not meeting this criterion were considered weakly associated with the exposure and subsequently excluded.

### 2.4 Mendelian randomization analysis

This study employed methods, including MR Egger, Weighted median, IVW, Simple mode, and Weighted mode, to identify the causal relationship between GM, metabolites, and asthma. IVW was the primary MR method used when horizontal pleiotropy was absent, and its results were considered the main indicator of causation. The correlation between GM, metabolites, and asthma was examined using the other four established MR methods. The MR-Egger intercept test was employed to identify any pleiotropy in the dataset, with a significance level of *p* < 0.05 indicating the presence of pleiotropy. Heterogeneity was assessed using IVW and MR-Egger tests, with *p* < 0.05 indicating significant heterogeneity. Additionally, a leave-one-out sensitivity analysis was conducted to determine the influence of specific genetic variants on the results, with scatter plots, forest plots, and funnel plots further illustrating the robustness and sensitivity of the findings.

### 2.5 Statistical analysis

All statistical analyses, encompassing MR and sensitivity checks, were conducted using R software. The MR analyses were performed with the “Two Sample MR” and “MR-PRESSO” software packages.

## 3 Results

### 3.1 Participants and genetic tool variables associated with gut microbiota and metabolites

**FIGURE 2:**
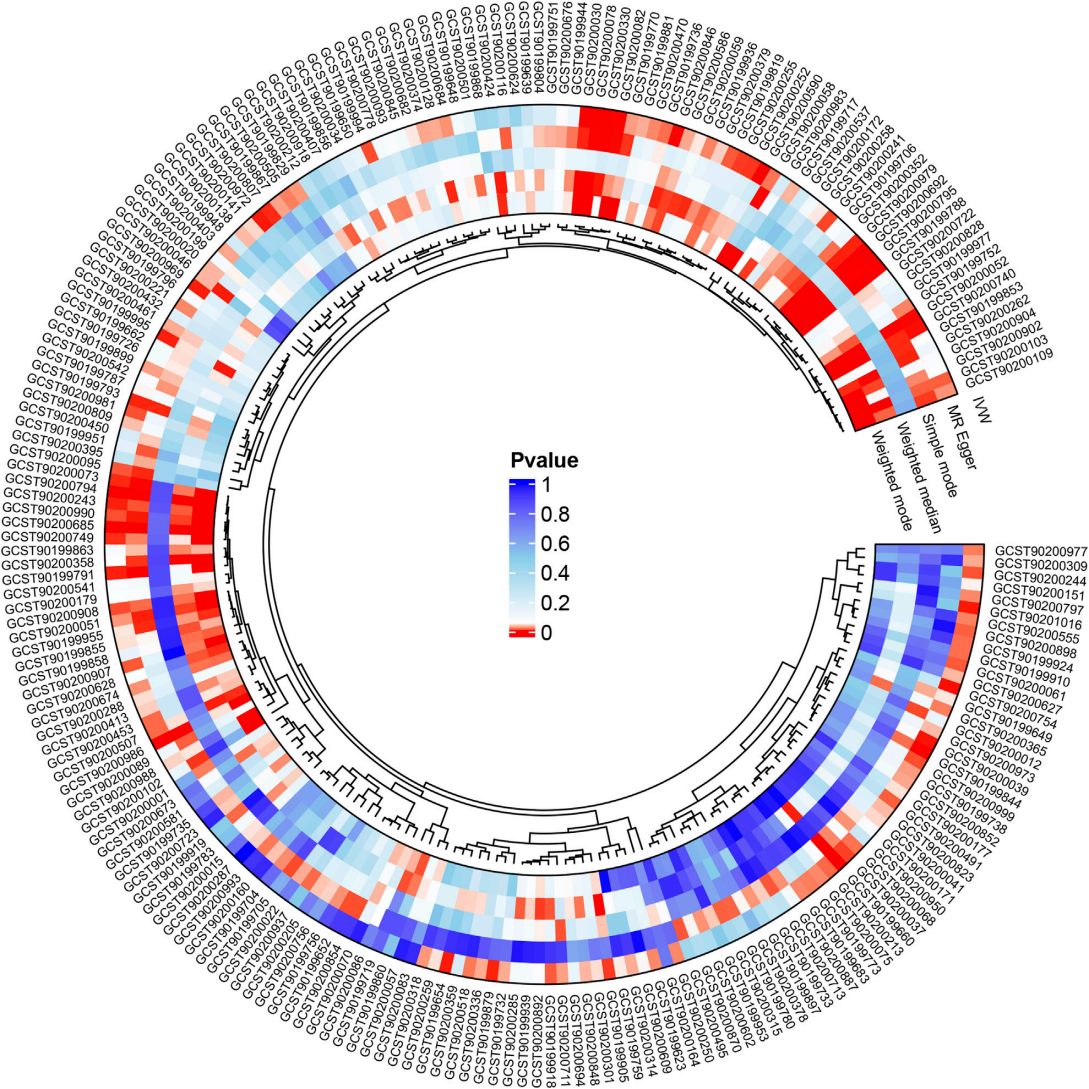
Following the integration of filtered plasma metabolites with asthma outcome data, Mendelian randomization analysis was performed using MR Egger, Weighted median, IVW, Simple mode, and Weighted mode methods, revealing 219 metabolites significantly linked to asthma (*p* < 0.05).

We refined the gut microbiota exposure data by applying a cut-off at F > 10 and eliminating deficient instrumental variables, which yielded 1531 SNP records (Supplementary Table S1). When we integrated this data with asthma outcome information, we identified seven bacterial species significantly associated with asthma (*p* < 0.05), as illustrated in Table 1. Similarly, we analyzed metabolite exposure data using F > 10 and excluding weak instrument variables, resulting in 34,843 SNP entries (Supplementary Table S2). Combining these findings with asthma outcomes, we found 219 metabolites significantly associated with asthma (*p* < 0.05), as depicted in Figure 2.

**TABLE 1:** MR results of causal relationships between the gut microbiota and asthma, and its phenotypes risk.

Outcome	Exposure	Method	*p*	OR	SNP
Asthma	ChristensenellaceaeR.7group	IVW	0.00275	0.98653	11
Asthma	Prevotella7	IVW	0.00407	1.00505	12
Asthma	Bifidobacterium	IVW	0.01453	1.00799	15
Asthma	RuminococcaceaeUCG003	Weighted median	0.01586	1.01147	14
Asthma	CandidatusSoleaferrea	Weighted median	0.04144	0.99385	13
Asthma	Bifidobacterium	Weighted median	0.04488	1.00836	15
Asthma	Akkermansia	IVW	0.04761	0.99155	12

### 3.2 Mendelian randomization analysis of intestinal microbiota and asthma

**FIGURE 3:**
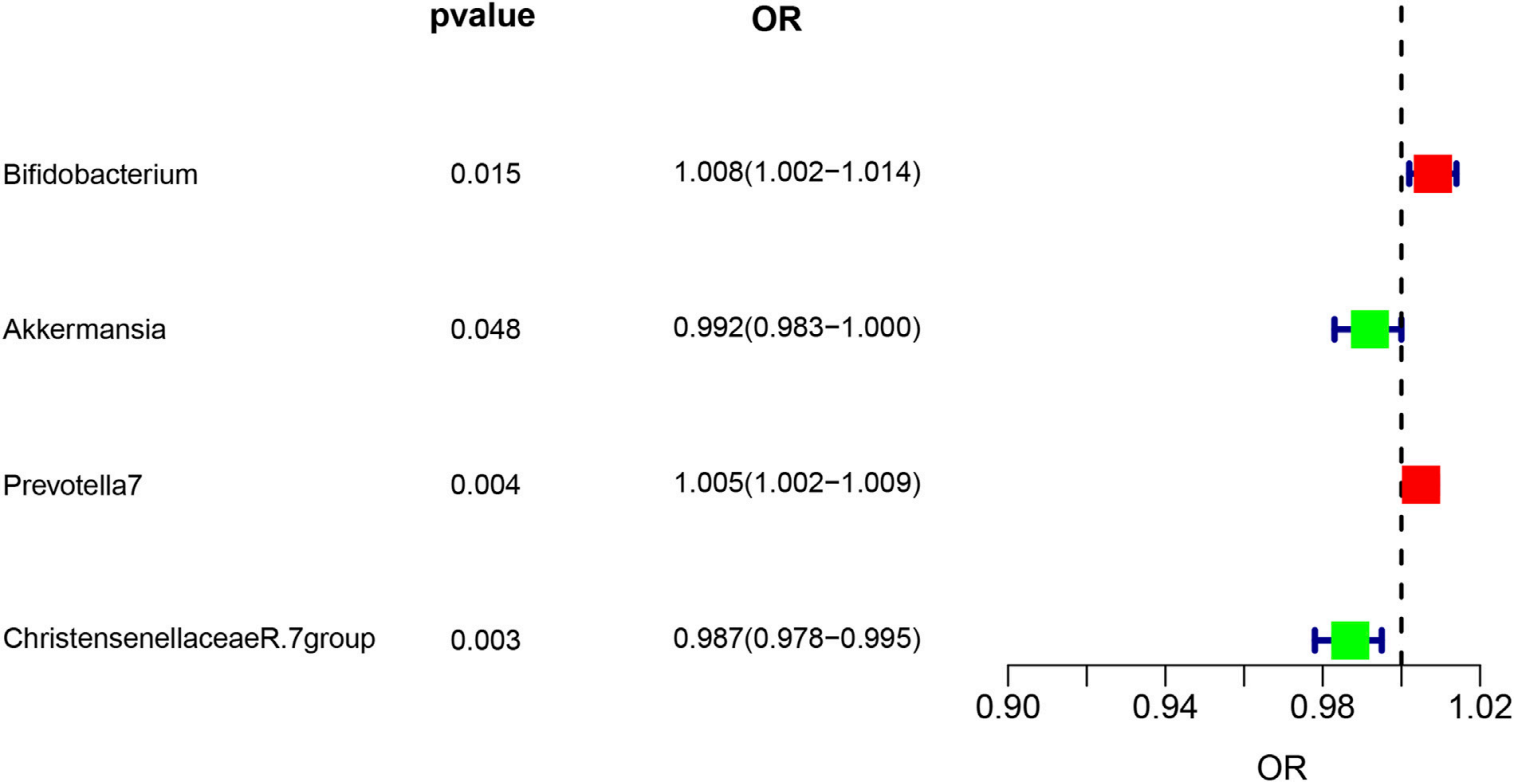
Forest plot illustrating the causal effect of Gut microbiota on asthma risk using the IVW method. Protective factors are depicted in green, while risk factors are shown in red.

Analysis of intestinal flora via the IVW method revealed certain correlations with asthma risk: Akkermansia and Christensenellaceae R.7 group bacteria demonstrated a negative correlation with asthma risk, suggesting a potential protective role. Conversely, Bifidobacterium and Prevotella7 bacteria showed a positive correlation, indicating they might act as risk factors for asthma (*p* < 0.05). These findings are illustrated in Figure 3.

### 3.3 Mendelian randomization analysis of 3 metabolites and asthma

**FIGURE 4:**
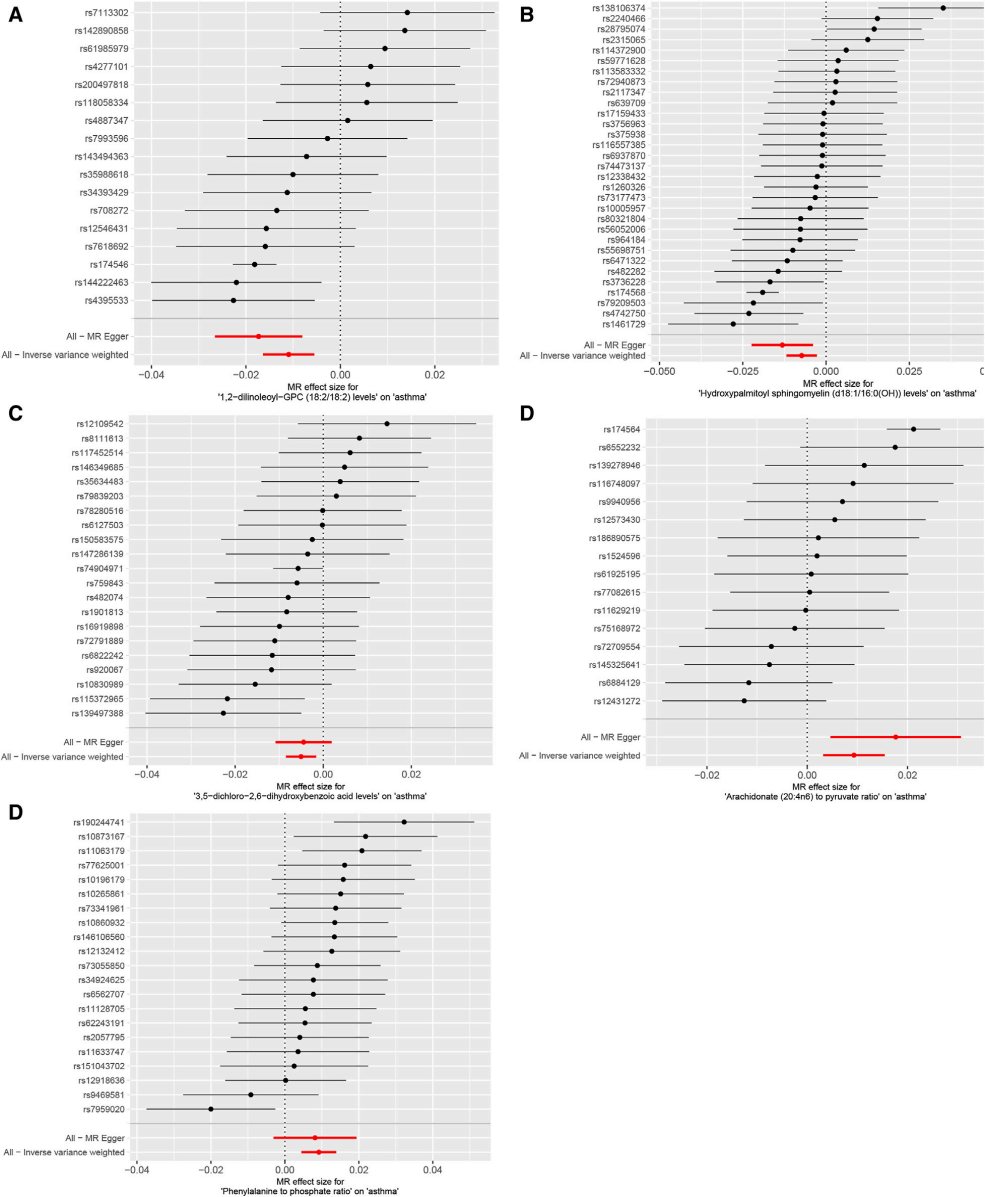
Mendelian randomization analysis visualizes the causal effects of plasma metabolites on asthma risk through forest plots. **(A)** The levels of 1,2-dilinoleoyl-GPC (18:2/18:2), **(B)** the levels of Hydroxypalmitoyl sphingomyelin [d18:1/16:0(OH)], **(C)** the levels of 3,5-dichloro-2,6-dihydroxybenzoic acid, **(D)** the ratio of Arachidonate (20:4n6) to pyruvate, **(E)** the ratio of Phenylalanine to phosphate. *p*-values less than 0.05 for all three methods: Weighted median, IVW, and Weighted mode methodologies.

The IVW analysis revealed that 14 were associated with asthma among the identified metabolites, with nine exhibiting negative correlations and five showing positive correlations (*p* < 0.05), as shown in Figure 4. The application of the Weighted median, IVW, and Weighted mode methods consistently produced significant *p*-values below 0.05. Particular metabolites such as 1,2-dilinoleoyl-GPC (18:2/18:2), Hydroxyalmitoyl sphingomyelin (d18:1/16:0(OH)), and 3,5-dichloro-2,6-dihydroxybenzoic acid displayed negative correlations with asthma. In contrast, metabolites Arachidronate (20: 4n6) to pyruvate ratio and Phenylalanine to phosphate ratio exhibited positive correlations with asthma, indicating potential risk factors (*p* < 0.05) (Figure 4), Reverse MR analysis did not reveal any correlation between asthma and metabolites.

### 3.4 Sensitivity analysis

**FIGURE 5:**
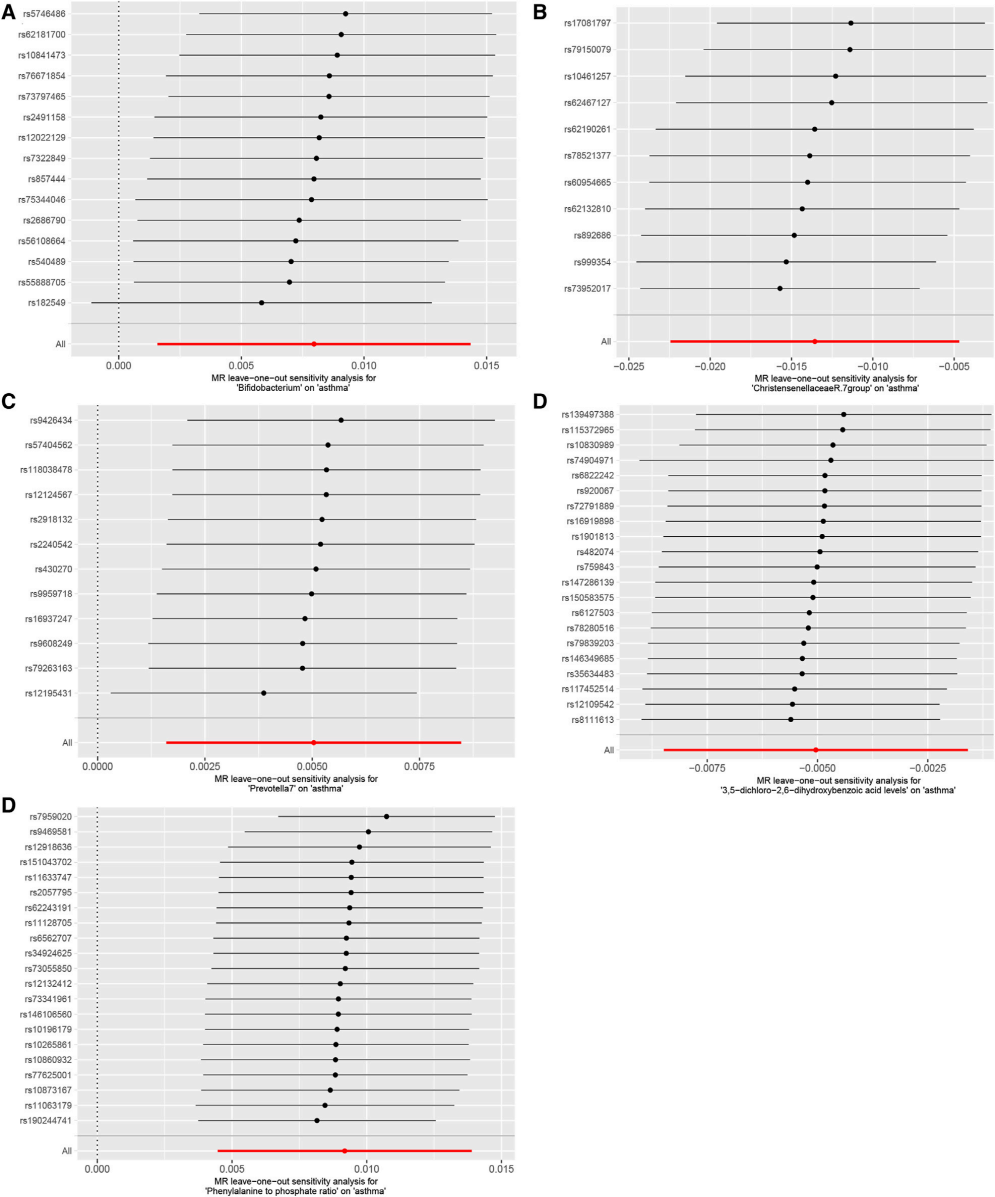
Using the leave-one-out method, the study analyzed the influence of the gut microbiome and blood plasma metabolites on asthma sensitivity, **(A)**. Bifidobacterium; **(B)**. Christensenellaceae R.7 group; **(C)**. Prevotella 7; **(D)**. 3,5-dichloro-2,6-dihydroxybenzoic acid levels; **(E)**. Phenylalanine to phosphate ratio. The results indicated that no individual SNP significantly impacted the overall effect.

Our Mendelian randomization study on the gut microbiome employed the IVW and MR-Egger methods to test heterogeneity. The results indicated no heterogeneity between Bifidobacterium, Christensenellaceae R.7 group, Prevotella 7, 3,5-dichloro-2,6-dihydroxybenzoic acid levels, and Phenylalanine to phosphate ratio SNP about the outcomes. However, heterogeneity was detected in the relationships involving Akkermansia, 1,2-dilinoleoyl-GPC (18:2/18:2) levels, Hydroxypalmitoyl sphingomyelin [d18:1/16:0(OH)] levels, and Arachidonate (20:4n6) to pyruvate ratio SNP with the outcomes (*p* > 0.05) (Table 2). The MR-Egger regression intercept did not show a significant deviation from zero, indicating no pleiotropy (*p* > 0.05) (Table 3). Furthermore, the leave-one-out sensitivity analysis demonstrated that the associations observed between the exposure factors and outcomes were not influenced by any single SNP (Figure 5).

**TABLE 2:** Analysis of the heterogeneity in genetic causal effects of gut microbiota and plasma metabolites.

Outcome	Exposure	Method	Q	Q_pval
Asthma	Akkermansia	MR Egger	20.30941	0.02646
Asthma	Akkermansia	IVW	22.42817	0.02126
Asthma	Bifidobacterium	MR Egger	18.30886	0.14614
Asthma	Bifidobacterium	IVW	19.06206	0.16259
Asthma	ChristensenellaceaeR.7group	MR Egger	11.70515	0.23045
Asthma	ChristensenellaceaeR.7group	IVW	13.50046	0.19702
Asthma	Prevotella7	MR Egger	6.287618	0.79055
Asthma	Prevotella7	IVW	8.393976	0.67764
Asthma	1,2-dilinoleoyl-GPC (18:2/18:2) levels	MR Egger	38.43188	0.00078
Asthma	1,2-dilinoleoyl-GPC (18:2/18:2) levels	IVW	45.19486	0.00013
Asthma	Hydroxypalmitoyl sphingomyelin (d18:1/16:0(OH)) levels	MR Egger	80.00606	0.00000
Asthma	Hydroxypalmitoyl sphingomyelin (d18:1/16:0(OH)) levels	IVW	85.53642	0.00000
Asthma	3,5-dichloro-2,6-dihydroxybenzoic acid levels	MR Egger	21.88672	0.28993
Asthma	3,5-dichloro-2,6-dihydroxybenzoic acid levels	IVW	21.94069	0.34374
Asthma	Arachidonate (20:4n6) to pyruvate ratio	MR Egger	39.96339	0.00026
Asthma	Arachidonate (20:4n6) to pyruvate ratio	IVW	45.64169	0.00006
Asthma	Phenylalanine to phosphate ratio	MR Egger	28.92686	0.06714
Asthma	Phenylalanine to phosphate ratio	IVW	28.98745	0.08801

**TABLE 3:** Multivariate analysis of the pleiotropic effects of gut microbiota and plasma metabolites on genetic causation.

Outcome	Exposure	Egger_intercept	Se	*p*
Asthma	Akkermansia	−0.00122	0.00119	0.33114
Asthma	Bifidobacterium	−0.00052	0.00071	0.47757
Asthma	ChristensenellaceaeR.7group	0.00118	0.00100	0.27018
Asthma	Prevotella7	−0.00218	0.00150	0.17733
Asthma	1,2-dilinoleoyl-GPC (18:2/18:2) levels	0.00095	0.00058	0.12505
Asthma	Hydroxypalmitoyl sphingomyelin (d18:1/16:0(OH)) levels	0.00086	0.00061	0.16747
Asthma	Arachidonate (20:4n6) to pyruvate ratio	−0.00148	0.00105	0.18025
Asthma	3,5-dichloro-2,6-dihydroxybenzoic acid levels	−0.00010	0.00047	0.83093
Asthma	Phenylalanine to phosphate ratio	0.00012	0.00061	0.84399

## 4 Discussion

Asthma is a common chronic inflammatory airway disease with complex aetiology, involving multiple factors such as genetics, environment, and immunity (Melissa, 2019; Hammad and Bart, 2021; Stevens et al., 2022). In recent years, the gut microbiome, as a key regulator of host health, has increasingly attracted attention for its relationship with asthma (Logo ń et al., 2023). This study investigated the causal relationship between gut microbiota, plasma metabolites, and asthma using a two-sample Mendelian randomization (MR) approach, combining extensive GWAS meta-analysis data from the MiBioGen consortium and GWAS summary statistics for 1,400 plasma metabolites from the NHGRI-EBI GWAS Catalog database. The results suggest that specific gut microbiota and plasma metabolites may have potential causal relationships with asthma, providing new research directions for asthma prevention and treatment.

Our research found that the abundance of Akkermansia and Christensenellaceae R.7 group were negatively associated with asthma risk, while Bifidobacterium and Prevotella 7 were positively associated with asthma risk. This finding is partially consistent with previous studies, but some discrepancies remain and require further investigation.

Akkermansia muciniphila, the major member of the Akkermansia genus, is a probiotic with significant benefits for host health (Derrien et al., 2004; Derrien et al., 2007; Zhang et al., 2019). Studies have shown that Akkermansia muciniphila can positively impact asthma by regulating gut barrier function, inhibiting inflammatory responses, and other mechanisms. Our research findings indicate that reduced levels of Akkermansia muciniphila may be associated with increased asthma risk, consistent with previous studies. For example, Esraah et al. found that resveratrol supplementation in asthma model mice significantly increased Akkermansia abundance and ameliorated asthma symptoms (Alharris et al., 2022). Additionally, David et al. demonstrated a negative correlation between asthma severity and faecal Akkermansia muciniphila levels (Michalovich et al., 2019). Although heterogeneity tests revealed high variability in Akkermansia muciniphila, it exhibited no pleiotropy. This variability could stem from differences in sequencing platforms, equipment, and operator procedures. Nonetheless, a literature review suggests a significant association between Akkermansia muciniphila and asthma. Our findings imply a potential genetic causal relationship between these gut microbes and asthma risk.

Christensenellaceae R.7 group is a gut microbiome associated with host health, involved in carbohydrate and protein metabolism, producing beneficial short-chain fatty acids, etc. (Morotomi et al., 2011; Demirci et al., 2019; Waters and Ruth, 2019). Chen et al. found that Christensenellaceae_R-7_group was associated with a lower risk of eczema, inhalant allergen sensitization, and physician-diagnosed inhalant allergy (Hu et al., 2021). Our findings suggest that Christensenellaceae R.7 group may positively impact asthma risk by modulating gut immunity and metabolism.

On the other hand, Bifidobacterium and Prevotella 7 were positively associated with asthma risk, suggesting a potential involvement in asthma pathogenesis. Previous studies have shown a protective role of Bifidobacterium in airway inflammation, but our findings contradict this. This may be attributed to the complex pathophysiology of asthma, where Bifidobacterium may play different roles in various asthma subtypes.

Prevotella is a gut microbiome associated with chronic inflammatory diseases, which can activate Toll-like receptor 2, induce Th17-mediated immune responses, and ultimately promote asthma development (Myxedema and conservative heart failure, 1970; Arumugam et al., 2011; Wu et al., 2011; Yadava et al., 2016).

Current research indicates that plasma metabolites may be involved in the development of asthma (Jung et al., 2013). Our study found that plasma metabolite levels of 1,2-dilinoleoyl-GPC (18:2/18:2), Hydroxypalmitoyl sphingomyelin [d18:1/16:0(OH)], and 3,5-dichloro-2,6-dihydroxybenzoic acid were negatively correlated with asthma, while the Arachidonate (20:4n6) to pyruvate ratio and Phenylalanine to phosphate ratio were positively correlated with asthma. These findings provide new insights into the metabolic mechanisms underlying asthma.

3,5-dichloro-2,6-dihydroxybenzoic acid, a potential participant in anti-inflammatory responses, may be produced by certain gut microbiota, which have been shown to generate anti-inflammatory metabolites such as short-chain fatty acids. Mirka et al. found that the intake of Arachidonate (20:4n6) could increase the risk of asthma in offspring (Lumia et al., 2011), and Sandra et al. also found its potential association with asthma development (Ekstr ö m et al., 2022). Our findings suggest that an elevated Arachidonate (20:4n6) to pyruvate ratio may be linked to an increased asthma risk.

Chih et al. revealed that highly sensitised asthma-related metabolites are primarily enriched in pyruvate and acetyl-CoA metabolism (Chiu et al., 2021). Cheryl et al. discovered that pyruvate kinase M2, which converts phosphoenolpyruvate to pyruvate, acts as a coactivator, enhancing the transcription of several pro-inflammatory cytokines (Van De Wettering et al., 2020). Numerous studies have also found that GM can regulate pyruvate metabolism (Cheng et al., 2022; Chen Qu et al., 2023; Xu et al., 2023). Our results suggest that an elevated Arachidonate (20:4n6) to pyruvate ratio might reflect abnormal pyruvate metabolism in asthma patients, thus exacerbating inflammatory responses.

Wei et al. found a significant increase in Phenylalanine levels when inducing allergic asthma in mice (Hsu et al., 2021), a finding supported by other researchers (Liu et al., 2018). It has also been established that GM can disrupt phenylalanine metabolism (Altered metabolome and microbiome features, 2023; Prognostic Value of Gut Microbe, 2023). After conducting heterogeneity tests, we noted that the levels of 1,2-dilinoleoyl-GPC (18:2/18:2), hydroxypalmitoyl sphingomyelin [d18:1/16:0 (OH)], and the arachidonate (20:4n6) to pulmonary ratio exhibit considerable heterogeneity, yet no pleiotropy was detected. A thorough literature review further confirms a strong link between these elements and asthma. Collectively, these findings suggest that GM may influence asthma by altering metabolomic pathways. Presently, knowledge about research on these metabolites remains limited, and studies are comparatively infrequent. More randomized clinical trials and functional experiments are necessary to confirm these findings and clarify the underlying mechanisms.

These findings further support the intricate interplay between gut microbiota, plasma metabolites, and asthma. Gut microbiota may influence host immune systems by modulating the production of metabolic products, contributing to the development of asthma. For instance, specific gut microbiota may suppress asthma-related inflammation by producing anti-inflammatory factors, while others may promote the production of inflammatory factors, exacerbating inflammation.

Our findings offer a novel perspective on understanding the pathophysiology of asthma and provide new avenues for future prevention and treatment strategies. For instance, modulating the composition and function of gut microbiota, through interventions like probiotic supplementation or faecal microbiota transplantation, could potentially improve asthma symptoms. Additionally, modifying dietary patterns to adjust metabolite levels could be explored as a preventive or therapeutic approach for asthma.

However, this study has several limitations. First, it is only an association study and cannot fully demonstrate causation. Future randomised controlled trials are needed to validate these findings. Second, the study only considered the relationship between gut microbiota, plasma metabolites, and asthma. However, changes in gut microbiota can be influenced by various factors such as dietary structure, environmental pollution, and medication use. These factors could affect the composition of gut microbiota and, consequently, the risk of developing asthma. Multi-factor analysis is required to comprehensively uncover the mechanisms underlying asthma development. Finally, the study sample was drawn from a European population, and the pathogenesis of asthma may vary across racial and geographic groups. The genetic background, living environment, and dietary habits of European populations may differ from those of other races, potentially limiting the generalisability of the findings. Future studies need to consider expanding the sample source, incorporating individuals from different racial and geographic groups, to validate the generalizability of the findings.

In conclusion, this study provides new evidence for understanding the causal relationship between gut microbiota, plasma metabolites, and asthma. Further research is needed to investigate the clinical significance of these findings and develop interventions targeting gut microbiota and plasma metabolites to enhance asthma prevention and treatment outcomes.

## 5 Conclusion

Our findings emphasize the potential role of gut microbiota and its metabolites in asthma pathogenesis, revealing new research directions and insights for innovative diagnostic, therapeutic, and prognostic approaches for this condition.

## 6 Data inclusion and exclusion criteria

SNPs associated with exposure factor categories reached genome-wide significance threshold (p < 1e-05). SNPs that did not meet the requirements were excluded based on LD analysis (R2< 0.001, clustering distance = 10,000 kb), using the 1,000 Genomes Project data for Europeans; a statistic F > 10 was set as the threshold for strong IVs, otherwise, IVs were considered to have a weak association with exposure and therefore excluded. IVs can only influence the outcome variable through the exposure factor.

## Data Availability

The datasets presented in this study can be found in online repositories. The names of the repository/repositories and accession number(s) can be found in the article/Supplementary Material.
